# Morphological Diversity and Diagnostic Evaluation of Thoracic Amyloidosis: Insights From a Tertiary Referral Center in Vietnam

**DOI:** 10.1155/pm/3849443

**Published:** 2026-05-05

**Authors:** Luong Dinh-Van, Hoang-Anh Nguyen-Cong, Phuong Duong-Minh, Bich-Ngoc T. Nguyen

**Affiliations:** ^1^ Lung Transplant Center, National Lung Hospital, Hanoi, Vietnam; ^2^ Center for Rare Lung Diseases and Respiratory Infections, National Lung Hospital, Hanoi, Vietnam; ^3^ Center for Pathology and Molecular Biology, National Lung Hospital, Hanoi, Vietnam; ^4^ University of Medicine and Pharmacy, Vietnam National University, Hanoi, Vietnam, vnu.edu.vn

**Keywords:** biopsy, calcification, computed tomography, thoracic amyloidosis

## Abstract

**Background:**

Thoracic amyloidosis is a rare disorder that often mimics malignancy or chronic infections. In tuberculosis‐endemic regions, diagnostic confusion frequently leads to unnecessary empiric treatments. This study was aimed at describing the clinical and radiological characteristics of thoracic amyloidosis and evaluating the safety profile of diagnostic biopsy procedures.

**Methods:**

We retrospectively analyzed 19 patients with histopathologically confirmed thoracic amyloidosis at the National Lung Hospital, Hanoi, Vietnam, between 2022 and 2025. Clinical symptoms, high‐resolution computed tomography (CT) features, and biopsy outcomes were recorded.

**Results:**

The mean age was 55 ± 16 years. Common symptoms included cough (*n* = 15, 88.2%) and dyspnea (*n* = 9, 52.9%). Isolated airway involvement was the most frequent phenotype (*n* = 6, 31.6%), followed by isolated nodular (*n* = 3, 15.8%) and diffuse alveolar septal (*n* = 2, 10.5%) patterns. Mixed radiological patterns were observed in five patients (26.3%), while isolated extrapulmonary thoracic involvement accounted for the remaining three patients (15.8%). Notably, isolated cystic disease was absent. Among the cases with nodular involvement (*n* = 5), 80.0% (*n* = 4) exhibited spiculated margins, mimicking malignancy. Calcification was a frequent finding, present in 14/19 cases (73.7%). CT‐guided transthoracic needle biopsy (TTNB) and endobronchial biopsy (EBB) achieved diagnostic yields of 87.5% (7/8) and 69.2% (9/13), respectively, with no major complications, suggesting they are generally well‐tolerated. All collected specimens were negative for *Mycobacterium tuberculosis*, reducing the likelihood of active pulmonary tuberculosis.

**Conclusions:**

Thoracic amyloidosis presents with diverse and often overlapping radiological patterns. Isolated nodular lesions frequently exhibit spiculated margins, posing a diagnostic challenge. However, the high prevalence of calcification and the acceptable safety profile of EBB and TTNB facilitate definitive diagnosis. In endemic areas, early tissue diagnosis is essential to differentiate amyloidosis from tuberculosis and avoid inappropriate therapy.

## 1. Introduction

Amyloidosis is a disorder characterized by the extracellular deposition of insoluble abnormal proteins as amyloid fibrils. A definitive diagnosis requires histopathological examination, specifically the identification of apple‐green birefringence under polarized light after Congo red staining [1, 2]. Respiratory amyloidosis is a rare condition that presents either as a localized disease or systemic involvement [3, 4]. Based on anatomical and radiological features, thoracic amyloidosis is classified into four main phenotypes: tracheobronchial, nodular, cystic, and diffuse alveolar septal [3, 5].

The clinical presentation of thoracic amyloidosis is nonspecific. Patients typically report chronic respiratory symptoms such as cough and dyspnea, which often delay the diagnosis [6, 7]. On computed tomography (CT), respiratory amyloidosis shows diverse manifestations. Nodular lesions with spiculated margins can resemble primary or metastatic lung malignancies [8, 9]. Lesions with calcifications or cystic changes may be mistaken for chronic granulomatous infections, such as tuberculosis, which is highly endemic in regions like Vietnam [5, 10]. While classical literature describes isolated phenotypes, mixed radiological patterns in individual patients are observed in clinical practice but remain underreported [11].

Although CT features can suggest the disease, tissue biopsy is required for a definitive diagnosis [12]. In practice, invasive procedures such as CT‐guided transthoracic needle biopsy (TTNB) or endobronchial biopsy (EBB) are sometimes delayed due to concerns about bleeding risks associated with amyloid‐infiltrated tissues [13]. These delays can result in unnecessary surgical resections or empiric treatments, including antituberculosis therapy.

This study describes the clinical and radiological characteristics of thoracic amyloidosis, focusing on mixed phenotypes and calcification patterns. In addition, it evaluates the diagnostic yield and complication rates of routine biopsy procedures to provide evidence for early histological evaluation in patients with chronic respiratory symptoms and compatible CT findings.

## 2. Methods

### 2.1. Study Design and Setting

This was a retrospective, cross‐sectional descriptive study conducted at the National Lung Hospital (Hanoi, Vietnam) between January 2022 and December 2025. The study protocol was reviewed and approved by the Institutional Review Board of the National Lung Hospital. Given the retrospective nature of the study, the requirement for informed patient consent was waived by the ethics committee.

### 2.2. Study Population

Patients were included in the study if they met the following criteria: admitted with respiratory symptoms or abnormal chest imaging and had a definitive histopathological diagnosis of respiratory amyloidosis. The definitive diagnosis was established based on the presence of characteristic amyloid deposits on biopsy specimens, confirmed by Congo red staining demonstrating apple‐green birefringence under polarized light.

### 2.3. Data Collection and Radiological Assessment

Demographic data, clinical presentations, and laboratory findings were extracted from the hospital′s electronic medical records. Details regarding diagnostic procedures, specifically EBB and CT‐guided TTNB, alongside their associated complications, were also recorded. The choice of biopsy modality was tailored to the anatomical location of the lesions: Central airway abnormalities were approached via bronchoscopy, whereas peripheral parenchymal nodules or consolidations were sampled using CT‐guided TTNB.

Baseline chest CT images were systematically and independently reviewed by two radiologists to evaluate radiological characteristics. Any discrepancies between the two reviewers were resolved through consensus discussion; if a consensus could not be reached, a third senior radiologist was consulted to make the final decision. The lesions were classified into specific phenotypes: tracheobronchial, nodular, cystic, diffuse alveolar septal, or mixed pattern. Detailed morphological features, including margin characteristics and calcification patterns, were meticulously documented.

Given the high tuberculosis burden in Vietnam, microbiological screening for *Mycobacterium tuberculosis* is a mandatory component of the initial diagnostic workup for all undiagnosed thoracic lesions at our institution. Concurrently with obtaining tissue for histopathological examination, specimens were routinely sent for microbiological evaluation. Specifically, bronchoalveolar lavage fluid was collected during bronchoscopy, and portions of tissue specimens obtained via transthoracic lung biopsy or extrapulmonary biopsy were routinely allocated for *M. tuberculosis* culture and smear.

### 2.4. Statistical Analysis

All statistical analyses were performed using Jamovi software, Version 2.6 (The Jamovi Project, 2025). Continuous variables were expressed as mean ± standard deviation (SD) or median with interquartile range (IQR), depending on the normality of the data distribution. Categorical variables were presented as frequencies and percentages.

## 3. Results

### 3.1. Baseline Clinical Characteristics

The demographic and baseline clinical characteristics of the 19 patients are summarized in Table 1. The mean age of patients was 55 ± 16 years, with a female proportion of 57.9% (11/19). The median duration from symptom onset to clinical presentation was 60 days (IQR: 30–150). The most frequent respiratory symptoms included cough (88.2%) and dyspnea (52.9%), followed by cachexia (35.3%) and chest discomfort (29.4%). None of the patients reported hemoptysis. The median white blood cell count was 6.9 G/L (IQR: 5.8–8.5), and the median C‐reactive protein (CRP) level was 3.7 mg/L (IQR: 1.6–16.2).

**Table 1:** General characteristics and chest CT findings.

Characteristics	Total (*n* = 19)
Mean age (years)	55 ± 16
Male, *n* (%)	8 (42.1)
Comorbidities, *n* (%)	9 (47.4)
Median duration of symptoms [IQR] (days)	60 [30–150]
Fever^a^, *n* (%)	4 (23.5)
Cachexia^a^, *n* (%)	6 (35.3)
Enlarged lymph nodes^a^, *n* (%)	3 (17.6)
Cough^a^, *n* (%)	15 (88.2)
Sputum production^a^, *n* (%)	3 (17.6)
Dyspnea^a^, *n* (%)	9 (52.9)
Chest discomfort^a^, *n* (%)	5 (29.4)
Hemoptysis^a^, *n* (%)	0 (0.0)
Median white blood cell count [IQR] (G/L)	6.9 [5.8–8.5]
Mean neutrophil count (G/L)	3.9 ± 1.2
Mean lymphocyte count (G/L)	2.1 ± 0.8
Mean hemoglobin value (G/L)	126.7 ± 16.8
Mean platelet count (G/L)	297.9 ± 100.2
Median CRP [IQR] (mg/L)	3.7 [1.6–16.2]
Micronodule, *n* (%)	1 (5.3)
Macronodule, *n* (%)	7 (36.8)
Mass, *n* (%)	2 (10.5)
Consolidation, *n* (%)	5 (26.3)
Ground‐glass opacity, *n* (%)	2 (10.5)
Cyst, *n* (%)	5 (26.3)
Septal thickening, *n* (%)	2 (10.5)
Cavity, *n* (%)	0 (0.0)
Bronchiectasis, *n* (%)	2 (10.5)
Tracheal thickening, *n* (%)	8 (42.1)
Bronchial thickening, *n* (%)	5 (26.3)
Calcification, *n* (%)	14 (73.7)
Pleural thickening, *n* (%)	1 (5.3)
Pleural effusion, *n* (%)	4 (21.1)
Mediastinal lymphadenopathy, *n* (%)	6 (31.6)

^a^Clinical symptom data were incomplete for two out of 19 patients.

### 3.2. Radiological Phenotypes and Morphological Features

Based on high‐resolution CT findings, the 19 patients were classified into three main categories: isolated intrapulmonary (*n* = 11, 57.9%), mixed patterns (*n* = 5, 26.3%), and isolated extrapulmonary thoracic involvement (*n* = 3, 15.8%). The detailed distribution of these radiological phenotypes is summarized in Table 2. Within the isolated intrapulmonary group, airway involvement was the most common pattern. Notably, isolated cystic amyloidosis was absent. Cystic lesions exclusively co‐occurred with other abnormalities (*n* = 4), including airway thickening, nodules, or mediastinal lymphadenopathy. Another mixed pattern consisted of concurrent nodular and airway involvement (*n* = 1).

**Table 2:** Distribution of thoracic amyloidosis phenotypes.

Classification	Radiological phenotypes	*n*(%)
Isolated intrapulmonary phenotype (*n* = 11, 57.9%)	Airway involvement	6 (31.6%)
	Nodular involvement	3 (15.8%)
	Diffuse alveolar septal	2 (10.5%)
Mixed phenotype (*n* = 5, 26.3%)	Cystic‐mixed with airway	2 (10.5%)
	Cystic‐mixed with nodular	1 (5.3%)
	Cystic‐mixed with mediastinal lymphadenopathy	1 (5.3%)
	Nodular‐mixed with airway	1 (5.3%)
Isolated extrapulmonary phenotype (*n* = 3, 15.8%)	Mediastinal lymphadenopathy	2 (10.5%)
	Pleural involvement	1 (5.3%)
Total		19 (100%)

The morphological characteristics of the four primary types of abnormalities observed on CT—airway, nodular, cystic, and diffuse alveolar septal—are described below. These descriptions characterize the individual nature of each lesion type, which may coexist in patients with mixed patterns. Specific morphometric measurements for detectable airway (*n* = 7) and nodular (*n* = 5) lesions are summarized in Table 3.

**Table 3:** Morphological characteristics of airway (*n* = 7) and nodular (*n* = 5) manifestations on CT.

Findings	Airway manifestations (*n* = 7)
Mean length (mm)	68.1 ± 34.3
Mean thickness (mm)	3.3 ± 2.3
Mean perimeter (mm)	47.9 ± 36.3
Calcification pattern	
Punctate, *n* (%)	3 (42.9)
Amorphous, *n* (%)	4 (57.1)
Popcorn, *n* (%)	0 (0.0)
Findings	Nodular manifestations (*n* = 5)
Number of nodules > 10, *n* (%)	2 (40.0)
Median amount of lobes affected [IQR]	3 [2–5]
Margin	
Smooth, *n* (%)	0 (0.0)
Lobulated, *n* (%)	1 (20.0)
Spiculated, *n* (%)	4 (80.0)
Median minimal diameter [IQR] (mm)	8 [4–8]
Median maximal diameter [IQR] (mm)	13 [10–22]
Peripheral predominance, *n* (%)	4 (80.0)
Lower lobe predominance, *n* (%)	4 (80.0)
Calcification pattern	
Punctate, *n* (%)	2 (40.0)
Amorphous, *n* (%)	0 (0.0)
Popcorn, *n* (%)	2 (40.0)

*Note:* Airway measurements were performed on seven out of nine patients with histological airway involvement (two patients had no visible CT abnormalities). The nodular group (*n* = 5) includes three isolated nodular cases, one cystic‐mixed nodular case, and one nodular‐mixed airway case.

#### 3.2.1. Airway Involvement

**Figure 1:**
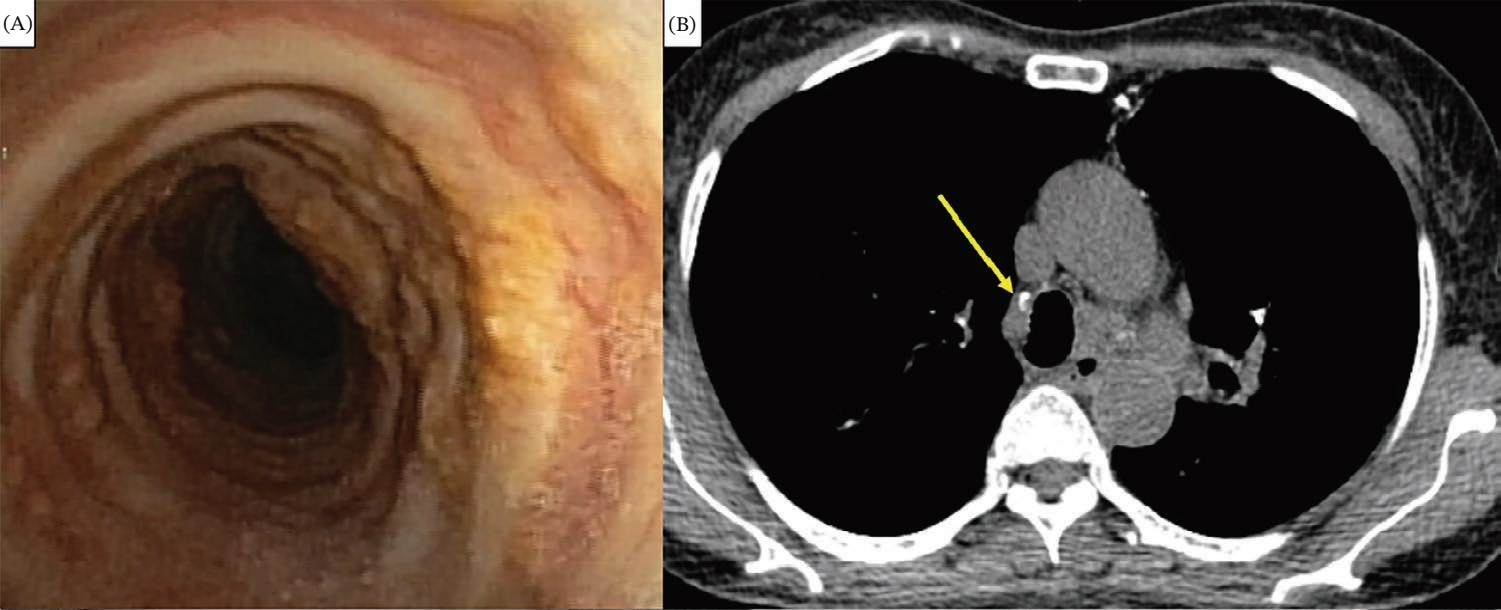
Tracheobronchial amyloidosis. (A) Bronchoscopic view showing irregular mucosal infiltration and luminal narrowing. (B) Axial chest CT (mediastinal window) demonstrates diffuse tracheal wall thickening accompanied by punctate calcification (yellow arrowhead).

Among the nine patients with histological airway involvement, seven patients exhibited detectable CT abnormalities, including wall thickening, luminal narrowing, and surface irregularity (Figure 1). The mean wall thickness was 3.3 ± 2.3 mm, with a mean longitudinal involvement length of 68.1 ± 34.3 mm.

#### 3.2.2. Diffuse Alveolar Septal Pattern

**Figure 2:**
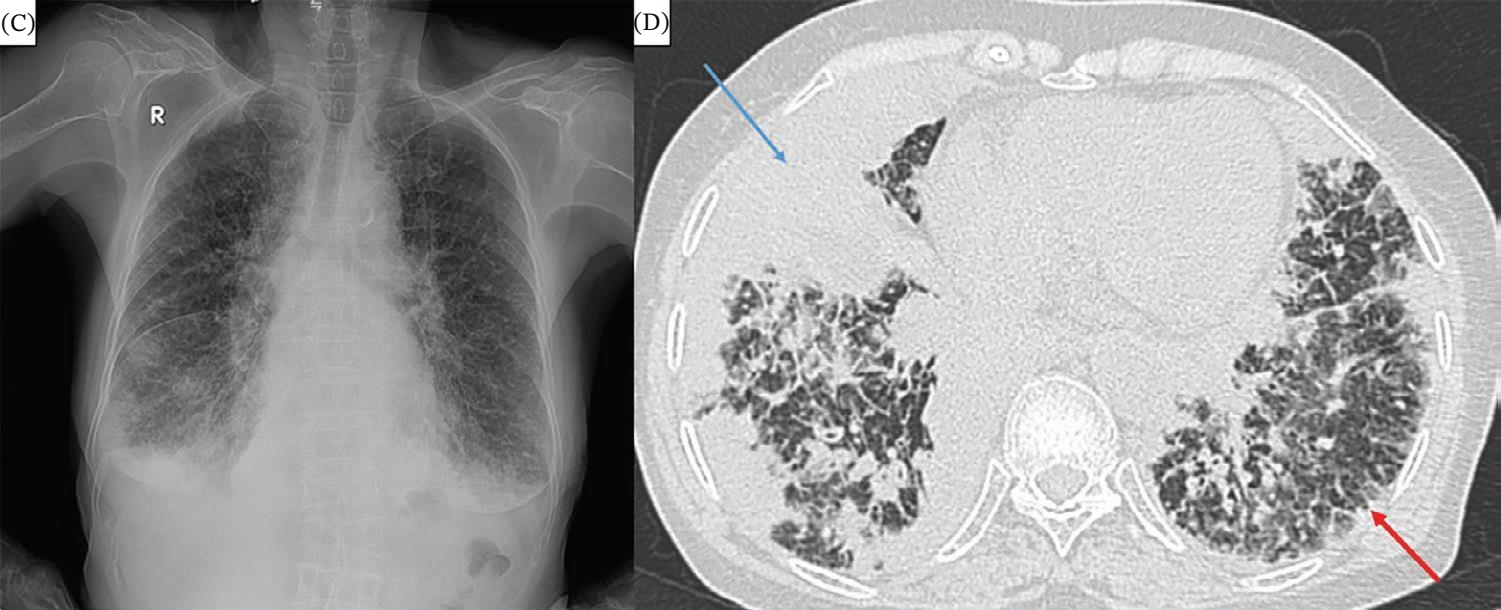
Diffuse alveolar septal amyloidosis. (C) Chest x‐ray revealing bilateral reticular opacities and infiltration with peripheral and lower lobe predominance. (D) Axial chest CT of the same patient demonstrating corresponding consolidation (blue arrow) and interlobular septal thickening (red arrow).

Two patients presented with this pattern, characterized by bilateral reticular opacities, consolidation, and septal thickening with a marked lower lobe predominance (Figure 2).

#### 3.2.3. Nodular Lesions

**Figure 3:**
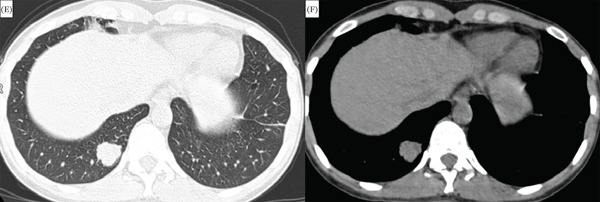
Nodular amyloidosis. (E) Axial chest CT (lung window) displays a solid parenchymal nodule with irregular margins in the right lower lobe. (F) Mediastinal window of the same lesion.

In five patients with nodular manifestations (comprising three isolated and two mixed cases), 80.0% (*n* = 4) exhibited spiculated margins, and 80.0% (*n* = 4) showed a predominance in the peripheral and lower lobes (Figure 3). The median maximal diameter of these nodules was 13 mm (IQR: 10–22 mm).

#### 3.2.4. Cystic Changes

**Figure 4:**
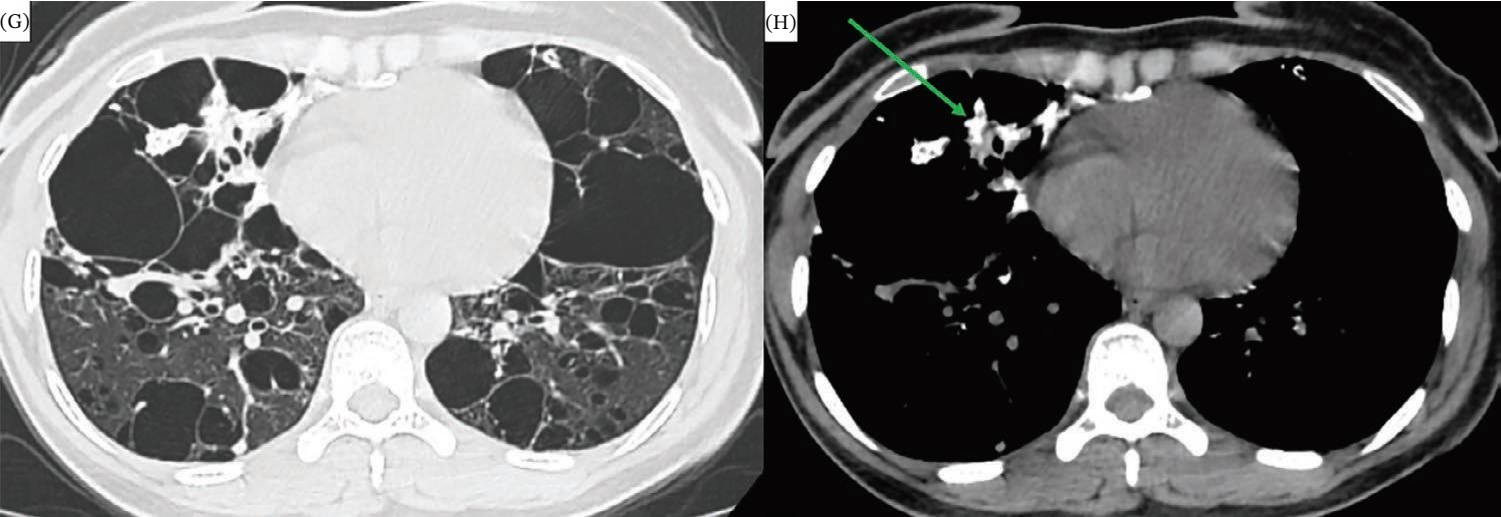
Cystic amyloidosis. (G) Axial chest CT (lung window) showing multiple thin‐walled cysts causing extensive parenchymal destruction. (H) Mediastinal window demonstrating a calcified nodule adjacent to the cysts (green arrow), representing a mixed radiological pattern.

All four patients with cystic manifestations (all of whom had mixed patterns) demonstrated multiple thin‐walled cysts (< 1 mm) with smooth margins (Figure 4). These cysts were typically distributed across all lung lobes without a specific zonal predilection.

Calcification was detected in 14 of the 19 patients (73.7%). Of these 14 cases, 13 exhibited calcifications within the lung parenchyma or airways. Among this subgroup (*n* = 13), the observed calcification patterns were punctate (*n* = 7), amorphous (*n* = 4), and popcorn‐like (*n* = 2). The remaining one patient (1/14) exhibited macroscopic popcorn‐like calcification localized within a mediastinal lymph node.

### 3.3. Diagnostic Yield and Complications of Biopsy Procedures

In our cohort, eight out of 19 patients underwent CT‐guided TTNB. The procedure confirmed thoracic amyloidosis in seven cases, yielding a diagnostic rate of 87.5%. Complications associated with TTNB included mild pneumothorax in two patients (25.0%) and mild pulmonary hemorrhage in two patients (25.0%). All complications were managed conservatively. EBB via bronchoscopy was performed in 13 patients, resulting in a confirmed diagnosis of tracheobronchial amyloidosis in nine cases (diagnostic yield: 69.2%). No major procedure‐related complications, such as severe bleeding or respiratory failure, were recorded. In all four patients presenting with cystic lesions, the histopathological diagnosis was established by biopsying the concurrent noncystic abnormalities (airway mucosa, pulmonary nodule, or enlarged lymph node) rather than the cysts themselves. Among the three patients with extrapulmonary involvement, two presented with lymphadenopathy. One patient, who had both mediastinal and peripheral lymph node enlargement, was diagnosed via a cervical lymph node biopsy. The other patient presented with isolated mediastinal lymphadenopathy and was diagnosed using endobronchial ultrasound–guided transbronchial needle aspiration. The third extrapulmonary case was diagnosed by pleural biopsy via thoracoscopy. While some patients underwent multiple procedures to achieve a diagnosis, the final diagnostic confirmation for the cohort without any overlap was distributed as follows: EBB (*n* = 9), CT‐guided TTNB (*n* = 7), and extrapulmonary procedures (*n* = 3), explicitly accounting for all 19 patients. Moreover, histopathological examination confirmed the deposition of amyloid in all 19 patients. However, advanced biochemical subtyping of the amyloid fibrils and comprehensive clinical evaluations to determine the underlying etiology were not routinely performed in this study. In accordance with our initial diagnostic protocols, comprehensive microbiological evaluations were performed concurrently with histopathological sampling. All obtained samples, including bronchoalveolar lavage fluid, lung tissue, pleural fluid, and lymph nodes, were tested negative for *M. tuberculosis*.

## 4. Discussion

This is the first Vietnamese analysis describing morphological overlap and biopsy safety in a tuberculosis‐endemic setting. This study describes the clinical and radiological characteristics, as well as the diagnostic evaluation, of thoracic amyloidosis in 19 patients. The demographic data show a mean age of 55 years and a slight female predominance (57.9%). This demographic distribution aligns with recent reports from Asian and South American cohorts, which often demonstrate a higher female ratio compared to earlier Western series [6, 14, 15]. Consistent with previous literature, the clinical presentation in our study was chronic and nonspecific. Symptoms such as cough and dyspnea were common, leading to significant diagnostic delays, with a median presentation time of 60 days [16, 17].

While the literature often divides respiratory amyloidosis into isolated tracheobronchial, nodular, cystic, or diffuse alveolar septal patterns [3, 18], our findings emphasize the frequent overlap of these features. A mixed radiological pattern was observed in 21.1% of the patients. This overlap supports the concept that amyloidosis represents a continuous spectrum of protein deposition affecting different compartments of the respiratory tract simultaneously rather than strictly isolated phenotypes [19, 20].

Radiologically, differentiating nodular amyloidosis from primary or metastatic lung cancer remains a major challenge [21, 22]. In this study, among patients with nodular involvement (*n* = 5), 80.0% exhibited spiculated margins. Histologically, spiculated margins in amyloidomas often result from a localized fibrotic and lymphoplasmacytic inflammatory response extending into the surrounding interstitium, closely mimicking the desmoplastic reaction of malignant tumors [8, 23]. However, calcification was identified in 73.7% of the total cases. The presence of punctate or amorphous calcifications, resulting from dystrophic calcification secondary to the high affinity of amyloid fibrils for calcium, is an important feature that helps differentiate amyloid deposits from noncalcified malignant neoplasms [24, 25].

The cystic phenotype also presented specific radiological patterns. Isolated cystic amyloidosis was not observed in our study; cysts consistently co‐occurred with other structural abnormalities, such as nodules or airway thickening. This finding aligns with the hypothesis that amyloid‐associated cysts rarely develop independently. The pathogenesis of these cysts is thought to involve a check‐valve mechanism caused by amyloid deposition in the small airways or ischemic destruction of the alveolar septa due to vascular infiltration [26, 27]. Furthermore, normal chest CT findings do not rule out airway involvement. Two patients with endoscopically confirmed airway amyloidosis had no detectable abnormalities on the initial CT. This discordance occurs because early mucosal amyloid deposition may not cause sufficient thickening or luminal narrowing to be resolved on standard CT scans, highlighting the need for bronchoscopic evaluation in patients with unexplained chronic respiratory symptoms [28].

The definitive diagnosis of amyloidosis strictly requires histopathological confirmation [29, 30]. Historically, physicians have hesitated to perform invasive biopsies in suspected amyloidosis due to the perceived risk of severe hemorrhage. This concern largely originates from systemic light‐chain amyloidosis, which can be associated with acquired factor X deficiency and vascular fragility [31]. However, our data demonstrate that routine biopsy procedures in patients with predominantly localized pulmonary involvement are generally well‐tolerated and yield high diagnostic rates. CT‐guided TTNB achieved an 87.5% diagnostic yield. Although mild pneumothorax and minor hemorrhage occurred (25.0% each), no patient required chest tube drainage or blood transfusion. EBB via bronchoscopy confirmed the diagnosis in 69.2% of the tested cases without any major complications, consistent with previous studies showing the acceptable safety profile of transbronchial approaches [13, 14]. For patients with cystic amyloidosis, biopsying concurrent noncystic lesions (e.g., nodules, thickened airways, or enlarged lymph nodes) proved to be a feasible and effective diagnostic strategy, avoiding the high risk of pneumothorax associated with directly puncturing thin‐walled cysts.

Beyond histopathological confirmation, our initial diagnostic approach provided a crucial clinical benefit, which is the concurrent microbiological evaluation of both tissue specimens and bronchoalveolar lavage fluid. In tuberculosis‐endemic regions like Vietnam, radiological findings such as calcified nodules and cystic changes, coupled with chronic respiratory symptoms, are frequently attributed to active or prior granulomatous infections [5, 10]. This diagnostic overlap often leads to the initiation of empiric antituberculosis therapy, exposing patients to potential hepatotoxicity and further delaying the correct diagnosis. Securing early tissue and microbiological data from accessible lesions is therefore an essential diagnostic step. While our consistently negative microbiological results significantly reduce the likelihood of active pulmonary tuberculosis, we acknowledge that the possibility of false negatives in highly endemic settings must always be considered. Interpreted cautiously, this comprehensive approach helps to exclude concurrent infections, thereby avoiding unnecessary antimicrobial treatments or overly aggressive surgical resections [30].

This study has several limitations. First, it was designed primarily as a descriptive retrospective analysis conducted at a single tertiary referral center, which inherently limits external generalizability. The relatively small sample size reflects the rarity of thoracic amyloidosis but restricts the ability to perform robust inferential statistical analyses. Given the limited number of cases within each radiological phenotype, formal comparative statistical testing between subgroups was intentionally not performed to avoid overinterpretation of underpowered analyses. Second, detailed amyloid subtyping was not systematically available. The definitive diagnosis of amyloidosis has evolved significantly with the introduction of proteomic‐based subtyping. Identification of the specific precursor protein is important because the management of amyloid light‐chain amyloidosis (plasma cell‐directed) differs fundamentally from that of amyloid A amyloidosis (targeting the underlying inflammatory condition) or hereditary forms [32]. In our study, while Congo red staining and apple‐green birefringence confirmed the presence of amyloid deposits, advanced subtyping was not performed. As a specialized tertiary center for respiratory medicine, our current diagnostic focus remains on the aspects of thoracic diseases. The unavailability of sophisticated techniques such as mass spectrometry—the current gold standard for subtyping—represents a key limitation. This constraint not only impacts the etiological classification of our cohort but also limits the precision of the therapeutic guidance we can offer, a challenge often encountered in resource‐limited or organ‐specific tertiary settings. Finally, the retrospective design may introduce selection bias, particularly as patients were identified through histopathological confirmation, potentially excluding individuals managed conservatively or diagnosed elsewhere. Future multicenter prospective studies incorporating comprehensive amyloid subtyping and standardized diagnostic protocols are warranted to validate and expand upon these findings.

## 5. Conclusion

Thoracic amyloidosis is a rare disease with a chronic clinical course and diverse radiological manifestations. Airway involvement is the most common phenotype, frequently overlapping with parenchymal lesions to form mixed patterns. Isolated cystic amyloidosis is uncommon, as cysts typically co‐occur with other structural abnormalities. Calcification within lesions is a frequent radiological feature that aids in the differential diagnosis against malignancy. Both EBB and CT‐guided TTNB demonstrate high diagnostic yields and acceptable safety profiles. In patients presenting with compatible CT findings and chronic respiratory symptoms, early histological and microbiological evaluation through the biopsy of accessible lesions is an essential and generally well‐tolerated step for a definitive diagnosis, helping to exclude endemic infectious etiologies such as tuberculosis.

**Nomenclature**
CRP: C‐reactive protein
CT: computed tomography
EBB: endobronchial biopsy
TTNB: transthoracic needle biopsy

## Author Contributions

L.D‐V. and H‐A.N‐C. drafted the manuscript and performed the literature review. B‐N.T.N. conceptualized the study and supervised the project. P.D‐M. performed the histological analysis, interpreted the pathology results, and participated in the revision of the manuscript.

## Funding

No funding was received for this manuscript.

## Disclosure

All authors read and approved the final manuscript.

## Ethics Statement

This retrospective study was conducted in accordance with the principles outlined in the Declaration of Helsinki and its subsequent revisions and received approval from the Ethics Committee of National Lung Hospital (IRB00011052). The requirement for informed consent was waived due to the retrospective nature of the study.

## Conflicts of Interest

The authors declare no conflicts of interest.

## Data Availability

Anonymized data can be made available upon request. Requests should be addressed to Bich‐Ngoc T. Nguyen (ngocn4@hotmail.com).
